# *SlCNGC1* and *SlCNGC14* Suppress *Xanthomonas oryzae* pv. *oryzicola*-Induced Hypersensitive Response and Non-host Resistance in Tomato

**DOI:** 10.3389/fpls.2018.00285

**Published:** 2018-03-06

**Authors:** Xuan-Rui Zhang, You-Ping Xu, Xin-Zhong Cai

**Affiliations:** ^1^Institute of Biotechnology, College of Agriculture and Biotechnology, Zhejiang University, Hangzhou, China; ^2^Center of Analysis and Measurement, Zhejiang University, Hangzhou, China

**Keywords:** cyclic nucleotide-gated ion channel (CNGCs), *Xanthomonas oryzae* pv. *oryzicola*, PAMP-triggered immunity, non-host resistance, Ca^2+^ signaling

## Abstract

Mechanisms underlying plant non-host resistance to *Xanthomonas oryzae* pv. *oryzicola* (*Xoc*), the pathogen causing rice leaf streak disease, are largely unknown. Cyclic nucleotide-gated ion channels (CNGCs) are calcium-permeable channels that are involved in various biological processes including plant resistance. In this study, functions of two tomato *CNGC* genes *SlCNGC1* and *SlCNGC14* in non-host resistance to *Xoc* were analyzed. Silencing of *SlCNGC1* and *SlCNGC14* in tomato significantly enhanced *Xoc*-induced hypersensitive response (HR) and non-host resistance, demonstrating that both *SlCNGC1* and *SlCNGC14* negatively regulate non-host resistance related HR and non-host resistance to *Xoc* in tomato. Silencing of *SlCNGC1* and *SlCNGC14* strikingly increased *Xoc*-induced callose deposition and strongly promoted both *Xoc*-induced and flg22-elicited H_2_O_2_, indicating that these two *SlCNGCs* repress callose deposition and ROS accumulation to attenuate non-host resistance and PAMP-triggered immunity (PTI). Importantly, silencing of *SlCNGC1* and *SlCNGC14* apparently compromised cytosolic Ca^2+^ accumulation, implying that SlCNGC1 and SlCNGC14 function as Ca^2+^ channels and negatively regulate non-host resistance and PTI-related responses through modulating cytosolic Ca^2+^ accumulation. *SlCNGC14* seemed to play a stronger regulatory role in the non-host resistance and PTI compared to *SlCNGC1*. Our results reveal the contribution of CNGCs and probably also Ca^2+^ signaling pathway to non-host resistance and PTI.

## Introduction

Each pathogen has its own host range. Non-host resistance is triggered when a non-adapted pathogen attempts to infect a plant species outside of its host range. Thus, non-host resistance is widely occurring, durable and broad-spectrum to non-adapted pathogens and is highly potential to be exploited in crop resistance engineering (Schulze-Lefert and Panstruga, 2011; Senthil-Kumar and Mysore, 2013). It has been clear that plant non-host resistance utilizes both preformed and induced defense mechanisms and frequently elicites hypersensitive response (HR) (Mysore and Ryu, 2004; Senthil-Kumar and Mysore, 2013). The preformed mechanisms generally consist of plant physical and chemical barriers, including antibiotic compounds (Bednarek and Osbourn, 2009; Fan et al., 2011). The induced non-host resistance is elicited after the preformed defense is overcome. As observed for host resistance, pathogen-associated molecular pattern (PAMP)-triggered immunity (PTI) and effector-triggered immunity (ETI) are often initiated in this layer of defense (Niks and Marcel, 2009; Senthil-Kumar and Mysore, 2013). Efforts to identifiy non-host resistance-related genes resulted in the finding that some genes, such as *PLDδ, GOX, SGT1*, and *NHO1*, contribute to both host and non-host resistance in Arabidopsis (Lu et al., 2001; Maimbo et al., 2010; Rojas et al., 2012; Pinosa et al., 2013). Generally speaking, the mechanisms underlying plant non-host resistance are far from well understood.

*Xanthomonas oryzae* pv. *oryzae* (*Xoo*) and *X. oryzae* pv. *oryzicola* (*Xoc*) are two important pathovars of *X. oryzae*, causing bacterial blight and leaf streak diseases, respectively, in rice (*Oryza sativa*), which is a staple food in many countries and a model plant for cereal biology (Nino-Liu et al., 2006). It is notable that these two pathovars utilize distinct mechanisms to infect rice leaves. *Xoo* is a vascular pathogen that enters rice leaves via the hydathodes, while *Xoc* penetrates rice leaves mainly through stomata or wound sites and colonizes intercellular spaces in the mesophyll (Nino-Liu et al., 2006). During infection of their plant hosts, many strains secrete transcription activator-like (TAL) effectors, which enter the host cell nucleus and activate specific corresponding host genes at effector binding elements (EBEs) in the promoter (Boch et al., 2009; Moscou and Bogdanove, 2009). It has been established that host resistance to *Xoo* is mostly related to the action of TAL effectors, either by polymorphisms that prevent the induction of susceptibility (S) genes or by executor resistance (R) genes with EBEs embedded in their promoter, and that induce cell death and resistance (Bogdanove et al., 2010; Zhang et al., 2015). *Xoc* is known to suppress host resistance, and no host *R* gene has been identified against it (Cai et al., 2017). Compared with the host resistance (Zhang and Wang, 2013), non-host resistance to *Xoo* and *Xoc* is much less studied. We have previously identified seven genes required for non-host resistance to *Xoo* in *Nicotiana benthamiana*. Among them are a calreticulin and a peroxidase, indicating that oxidative burst and calcium-dependent signaling pathways play an important role in non-host resistance to *Xoo* (Li et al., 2012). However, molecular mechanisms underlying non-host resistance to *Xoc* remain largely unknown. Whether the oxidative burst and calcium signaling pathway contribute to the non-host resistance to *Xoc* as to *Xoo* awaits examination.

The cyclic nucleotide-gated ion channels (CNGCs) are suggested to be one of the important pathways for conducting Ca^2+^ ions in signaling transduction (Talke et al., 2003). They are ligand-gated Ca^2+^-permeable divalent cation-selective channels that are often localized in plasma membrane, presumptively are activated by direct binding of cyclic nucleotides and are complexly regulated by binding of calmodulin (CaM) to the CaM Binding (CaMB) domain (Chin et al., 2009; Ma et al., 2009; Wang et al., 2013; Gao et al., 2014; DeFalco et al., 2016a,b; Fischer et al., 2017). The plant CNGCs are involved in numerous biological functions varying from plant development and stress tolerance to disease resistance (Kaplan et al., 2007; Qi et al., 2010; Ma and Berkowitz, 2011; Moeder et al., 2011). CNGCs are well conserved among plant species, comprising 20 members in Arabidopsis and 18 members in tomato (Saand et al., 2015a,b). Earlier studies revealed that *AtCNGC2, AtCNGC4, AtCNGC11*, and *AtCNGC12* and their homologs play an important role in disease resistance against various pathogens (Yu et al., 1998; Clough et al., 2000; Balagué et al., 2003; Jurkowski et al., 2004; Yoshioka et al., 2006; Ali et al., 2007; Ma and Berkowitz, 2011; Chin et al., 2013; Fortuna et al., 2015; Saand et al., 2015a). Later, other *CNGC* members such as *SlCNGC1* and *SlCNGC14* were also found to contribute to disease resistance (Saand et al., 2015a,b). We found that *SlCNGC1* and *SlCNGC14* play a negative role in non-host resistance to *Xoo* in tomato (Saand et al., 2015b). However, whether these *SlCNGCs* indeed encode functional CNGC channels and whether and how they function in non-host resistance to *Xoc* in tomato remains further study.

Our data in this study strongly indicate that SlCNGC1 and SlCNGC14 function as Ca^2+^ channels and negatively regulate tomato non-host resistance to *Xoc* through modulating ROS accumulation and callose deposition. Our results provide evidence for the contribution of CNGC-mediated Ca^2+^ signaling pathway to non-host resistance.

## Materials and Methods

### Plant Growth and Inoculation

Tomato (cv. Money Maker) plants were grown in growth room at 21°C with16 h light/8 h dark photoperiod. For disease resistance evaluation analyses, tomato plants were inoculated with *X. oryzae* pv. *oryzicola* (*Xoc*). After single colony propagation culture in NA medium, the *Xoc* bacterial cells were collected and resuspended in ddH_2_O to 1 × 10^8^ cfu mL^-1^. The bacterial suspension was then infiltrated into leaves with sterilized needleless syringe. The infiltration zones were marked immediately after infiltration. The inoculated plants were grown in growth room at 26°C with16 h light/8 h dark photoperiod.

### Gene Silencing Analyses

The virus-induced gene silencing (VIGS) target fragments of SlCNGC1 (Solyc01g095770.2) and SlCNGC14 (Solyc03g114110.2) were amplified by RT-PCR with gene-specific primers (Supplementary Table S1) and ligated into the TRV-based VIGS vector pYL156, which was subsequently electroporated into *Agrobacterium tumefaciens* strain GV3101 for VIGS analyses. VIGS analyses were conducted with vacuum infiltration delivery approach as described using recombinant pYL156 with insertion of an eGFP fragment instead of an empty pYL156 as control to alleviate viral symptom (Saand et al., 2015a). At about 3 weeks post agro-infiltration, plants were inoculated with *Xoc* as described above.

### Detection of Callose Deposition

Callose deposition were stained with aniline blue and visualized in fluorescence microscope. Briefly, the collected leaves were washed twice with ddH_2_O and ethanol, respectively, and cleared in acetic acid-ethanol (1:3) for 4 h. After washed twice with ddH2O, the leaves were incubated in aniline blue solution [150 mM KH_2_PO_4_, 0.1% (w/v) aniline blue, pH 9.5] for 1 h. The stained leaves were washed with ddH_2_O and observed in 30% glycerol by fluorescence microscopy.

### Bacterial Number Counting Assays

Bacterial numbers in *Xoc*-infiltrated leaves of silenced plants were determined as previously reported (Li et al., 2012).

### Detection of ROS

*Xoc*-inoculated leaves of tomato plants were detached and stained with 3,3-diamino benzidine hydrochloride (DAB) (1mg/mL) as described (Li et al., 2015). Quantitative determination of PAMP-elicited H_2_O_2_ was conducted using a luminol-based approach (Saand et al., 2015a). For each experiment, six tomato leaf disks of 3 mm at diameter from three plants were dipped in distilled water in the light over night. The leaf disks were then transferred into 50 μL of distilled water in a 96-well plate. After addion of the same volume mixture which contains 200 nM luminol (Sigma-Aldrich), 20 μg of horseradish peroxidase and 200 nM flg22, H_2_O_2_ were measured for 35 min as luminescence using a Microplate Luminometer (Titertek Berthold, Germany).

### Calcium Assay

Transient increase of cytosolic Ca^2+^ concentration was monitored in the Aequarin transgenic tomato lines. For each experiment, six leaf disks were punched from three plants and vacuum-infiltrated in 12.5 μM coelenterazin h (Sigma) on a 96-well plate for 1 min and then set for 2 h at room temperature. Before measurement, the coelenterazin solution was gently removed and 200 μL of PAMP solution (100 nM flg22) was added to the wells. Luminescence was measured using a Microplate Luminometer (Titertek Berthold, Germany).

### Gene Expression Analyses by qRT-PCR

Five defense-related Ca^2+^ signaling genes SlCDPK10 (Solyc11g018610.1), SlCAMTA3 (Solyc04g056270.2), SlCBP60G (Solyc01g100240.2), SlCAM2 (Solyc10g081170.1) and SlCAM6 (Solyc03g098050.2) were subjected to gene expression analyses (Zhao et al., 2013; Rahman et al., 2016b; Wang et al., 2016). Total RNA was isolated by Trizol (TaKaRa, Japan) extraction according to the manufacture’s instructions. RNAs were treated with DNase I (TaKaRa, Japan) and then reverse transcribed. Quantitative real time PCR (qRT-PCR) was performed as previously described using the StepOne Real-Time PCR system (Applied Biosystems, United States) with SYBR Green PCR Master Mix (TaKaRa, Japan) (Saand et al., 2015a). 18s rDNA was used as internal control. The accession number and the specific primers for amplification of the analyzed genes were listed (Supplementary Table S1). The expression of target genes relative to control was calculated based on a value of 2^-ΔΔCt^ as recommended by the manufacturer. Meanwhile, semi-qRT-PCR for these genes were conducted in parallel and obtained PCR products were analyzed by agarose gel electrophoresis.

### Statistical Analyses of Data

All experiments were conducted three times independently. The quantitative measurement data were statistically analyzed using SPSS software and represent means ± standard error. Significant difference between mean values was determined with DMRT (*p* < 0.05).

## Results

### Silencing of *SlCNGC1* and *SlCNGC14* Enhanced *Xoc*-Induced Hypersensitive Response and Non-host Resistance

**FIGURE 1:**
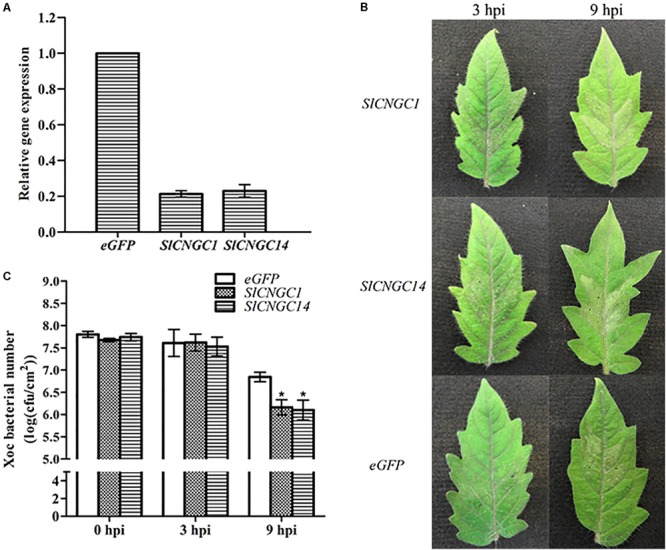
Silencing of *SlCNGC1* and *SlCNGC14* enhanced HR and non-host resistance to *Xoc* in tomato. **(A)** Silencing efficiency analysis. Plants infiltrated with *Agrobacterium* suspensions carrying an eGFP-control vector served as control plants. Accumulation level of *SlCNGC1* and *SlCNGC14* transcript in VIGS-treated plants and the eGFP-control plants was detected by qRT-PCR analyses. **(B)** *Xoc*-induced hypersensitive response (HR). Photographs were taken at 3 and 9 hpi. **(C)** *Xoc* bacterial number counting. *Xoc* bacterial numbers were counted in leaf areas that were inoculated with bacterium at 3 and 9 hpi. At least five leaves were tested for each treatment, and the data represent means ± standard error (SE). Significant differences between treatments and the control are indicated by an asterisk (*p* < 0.05).

In order to dissect the function of *SlCNGC1* and *SlCNGC14* in *Xoc*-induced HR and non-host resistance, we used TRV-based VIGS vector to perform VIGS analyses for these genes. Transcripts of *SlCNGC1* and *SlCNGC14* genes accumulated to only about 20% of the eGFP silencing control (**Figure 1A**), indicating that *SlCNGC1* and *SlCNGC14* genes had been effictiently silenced in these plants. Inoculation assays with *Xoc* in these silencing plants demonstrated that *SlCNGC1*- and *SlCNGC14*-silenced leaves showed more severe *Xoc*-induced HR than the eGFP control leaves. *SlCNGC1*- and *SlCNGC14*-silenced leaves showed obvious HR necrosis at 3 h post *Xoc* inoculation, and exhibited strong HR necrosis at 9 hpi, especially *SlCNGC14*-silenced leaves, which had turned into desiccative, while the eGFP control plants displayed barely visible and weak HR necrosis at 3 and 9 hpi, respectively (**Figure 1B**). This result indicated that silencing of *SlCNGC1* and *SlCNGC14* enhanced *Xoc*-induced HR.

We further counted *Xoc* bacterial number in the infiltrated leaf areas. The result showed that compared with the eGFP controls, *Xoc* bacterial number in *SlCNGC1*- and *SlCNGC14*-silenced plants decreased significantly by 0.7 orders of magnitude at 9 hpi (**Figure 1C**), indicating that silencing of *SlCNGC1* and *SlCNGC14* enhanced non-host resistance to *Xoc*.

Collectively, these results implied that both *SlCNGC1* and *SlCNGC14* might negatively regulate non-host resistance related HR cell death and non-host resistance to *Xoc* in tomato.

### Silencing of *SlCNGC1* and *SlCNGC14* Increased *Xoc*-Induced Callose Deposition

**FIGURE 2:**
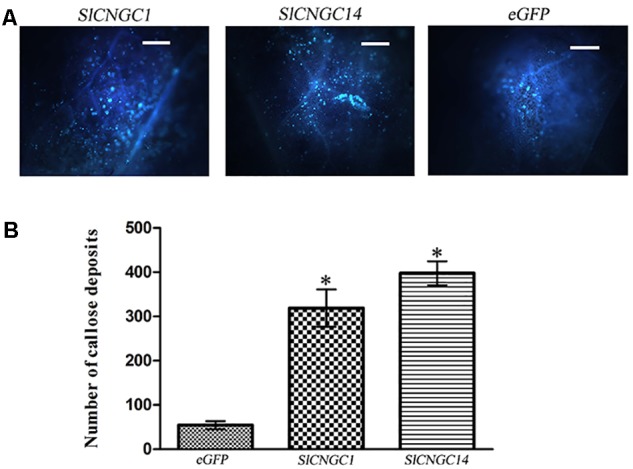
Silencing of *SlCNGC1* and *SlCNGC14* increased *Xoc*-induced callose deposition. Leaves inoculated with *Xoc* at 4 hpi were stained with aniline blue and visualized in fluorescence microscope. A typical staining result **(A)** and its quantification result **(B)** were shown. Significant differences between treatments and the control are indicated by an asterisk (*p* < 0.05).

To probe the mechanisms underlying *SlCNGC1*- and *SlCNGC14*-dependent regulation of non-host resistance against *Xoc*, effect of these *CNGC* genes on callose deposition upon pathogen infection was examined. Callose deposition was visible in the aniline blue-stained leaves under the fluorescence microscopy. Microscopic observation result showed that *SlCNGC1*- and *SlCNGC14*-silenced leaves deposited much more callose than eGFP control leaves (**Figure 2A**). Further quantification revealed that callose deposits in *SlCNGC1*- and *SlCNGC14*-silenced leaves was 5.9- and 7.3-fold respectively, as many as in the eGFP control leaves at 4 hpi (**Figure 2B**). This result demonstrates that *SlCNGC1* and *SlCNGC14* repress the callose deposition upon pathogen infection to alleviate non-host resistance to *Xoc* in tomato.

### Silencing of *SlCNGC1* and *SlCNGC14* Promoted ROS Accumulation

**FIGURE 3:**
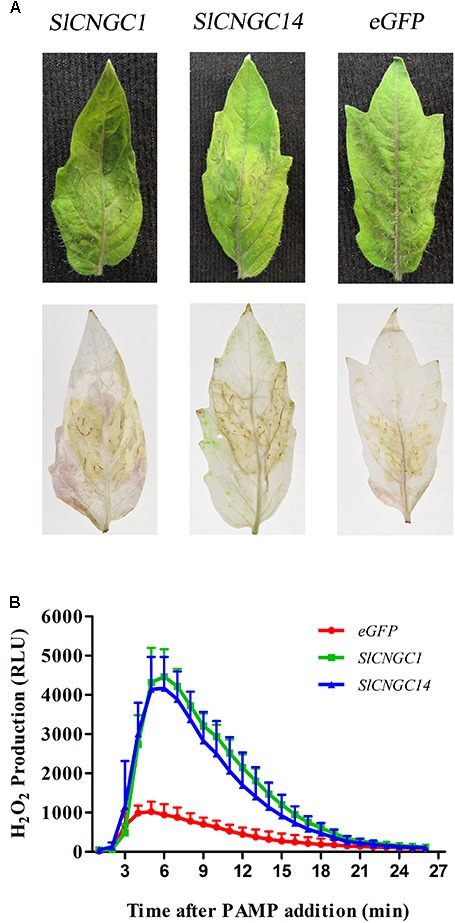
Silencing of *SlCNGC1* and *SlCNGC14* promoted *Xoc*-induced and flg22-elicited ROS accumulation. **(A)** *Xoc*-induced ROS assay. Hydrogen peroxide (H_2_O_2_) accumulation at 4 hpi was detected through Diaminobenzidine (DAB) staining analysis. The *Xoc*-infriltrated leaves before (Up panel) and after (Down panel) staining analyses were presented. **(B)** flg22-elicited ROS assay. Production of H_2_O_2_ induced by 100 nM flg22 was detected using a luminol-based assay in leaf disks of *SlCNGC1*-, *SlCNGC14*-silenced plants and the eGFP non-silenced control plants. Data are shown as relative luminol units (RLUs) and represent the mean ± SE of three independent experiments.

Reactive oxygen species (ROS) is indispensable to *X. oryzae* pv. *oryzae* (*Xoo*)-induced HR and non-host resistance (Li et al., 2015). Thus, we analyzed the effect of *SlCNGC1* and *SlCNGC14* on the production of hydrogen peroxide (H_2_O_2_), one of the primary species of ROS. DAB staining result demonstrated that infiltration areas of *SlCNGC1*- and *SlCNGC14*-silenced leaves showed significantly stronger DAB staining than those of control (**Figure 3A**), implying that silencing of *SlCNGC1* and *SlCNGC14* increased the production of H_2_O_2_.

Effect of *SlCNGC1* and *SlCNGC14* on the PAMP (flg22)-elicited H_2_O_2_ accumulation was further examined using leaf disk assays and was indicated as relative luminescence (RLU). The luminol-based assay showed that flg22-induced H_2_O_2_ in *SlCNGC1*- and *SlCNGC14*-silenced leaves culminated to over 4100 RLU, while that in eGFP control leaves was peaked only to 940 RLU (**Figure 3B**), demonstrating that silencing of *SlCNGC1* and *SlCNGC14* promoted the production of flg22-elicited H_2_O_2_.

Together, these results suggest that *SlCNGC1* and *SlCNGC14* supress the ROS accumulation to attenuate non-host resistance and PTI.

### Silencing of *SlCNGC1* and *SlCNGC14* Compromised Cytosol Ca^2+^ Influx

**FIGURE 4:**
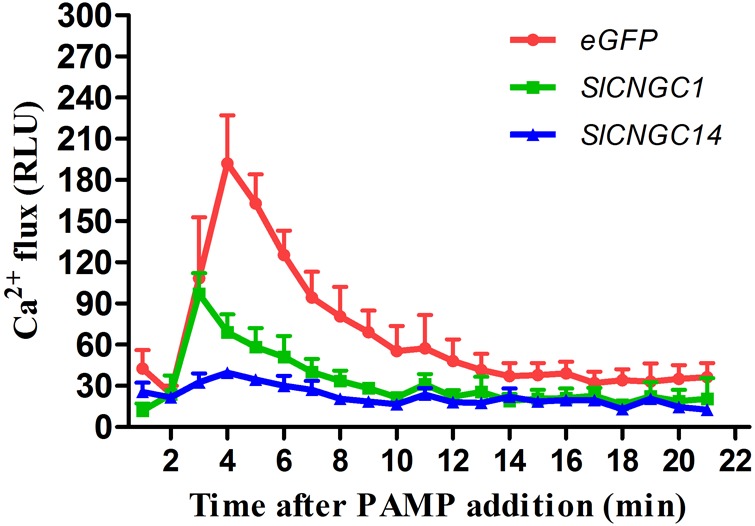
Silencing of *SlCNGC1* and *SlCNGC14* compromised cytosolic Ca^2+^ accumulation. Production of Ca^2+^ flux elicited by 100 nM flg22 was detected using a luminescence-based assay in leaf disks of *SlCNGC1*-, *SlCNGC14*-silenced plants and the eGFP non-silenced control plants of the aequorin transgenic tomato lines. Data are shown as relative luminescence units (RLU) and represent the mean ± SE of three independent experiments.

Some Arabidopsis CNGCs have been proved to be functional Ca^2+^ channels (Gao et al., 2014; Wang et al., 2017). To examine whether SlCNGC1 and SlCNGC14 function as Ca^2+^ channels, effect of silencing of these genes on accumulation of cytosolic Ca^2+^ elicited by the PAMP flg22 was monitored through leaf disk assays using aequorin transgenic tomato lines. In eGFP control aequorin transgenic plants, flg22-triggered Ca^2+^ increased rapidly and peaked to 192 RLU, while those in *SlCNGC1*- and *SlCNGC14*-silenced aequorin transgenic plants strongly decreased with the peak value drop to only 97 and 39 RLU, respectively (**Figure 4**). This result indicates that SlCNGC1 and SlCNGC14 function as Ca^2+^ channels and negatively regulate non-host resistance and PTI through modulating cytosolic Ca^2+^ accumulation.

### Silencing of *SlCNGC1* and *SlCNGC14* Altered Expression of Defense-Related Ca^2+^ Signaling Genes

**FIGURE 5:**
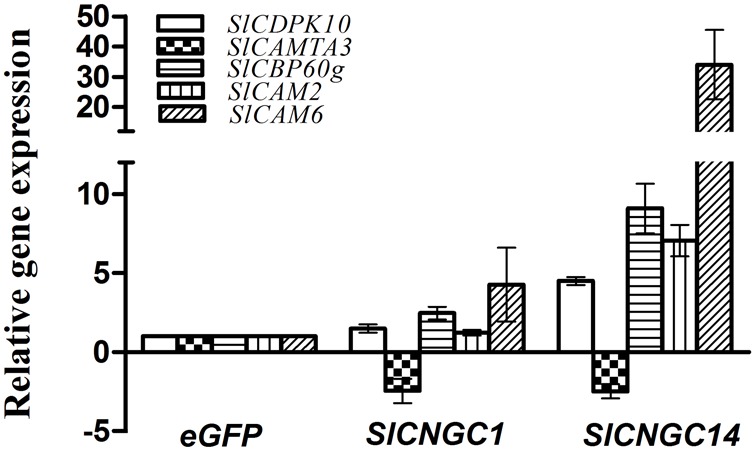
Silencing of *SlCNGC1* and *SlCNGC14* altered expression of defense-related Ca^2+^ signaling genes. Expression of defense-related Ca^2+^ signaling genes *SlCAM2, SlCAM6, SlCDPK10, SlCAMTA3*, and *SlCBP60g* were examined for *SlCNGC1*-, *SlCNGC14*-silenced plants relative to the eGFP non-silenced control plants after inoculation with *Xoc* at 4 hpi by qRT-PCR with 18s rDNA gene serving as a loading control gene. Data represent the mean ± SE of three independent experiments.

To obtain a clue to understand how *SlCNGC1* and *SlCNGC14* genes, as Ca^2+^ channel genes, regulate plant disease resistance, we examined effect of silencing of these genes on expression of a set of defense-related Ca^2+^ signaling genes to probe the possibility of their involvement in *SlCNGC1*- and *SlCNGC14*-mediated resistance regulation. The genes under this expression analysis included two CaM genes *SlCaM2* and *SlCaM6*, a tomato homolog (*SlCDPK10*) of Arabidopsis calcium-dependent protein kinase gene *AtCDPK11*, and the tomato homologs (*SlCBP60g* and *SlCAMTA3*) of two Arabidopsis CaM-binding transcription factors *AtCBP60g* and *AtCAMTA3*. All these genes play an important role in regulating disease resistance (Du et al., 2009; Wang et al., 2009, 2011; Boudsocq et al., 2010; Zhao et al., 2013; Sun et al., 2015; Rahman et al., 2016a,b; Wang et al., 2016). Result of qRT-PCR showed that at 4 h after inoculation with *Xoc, SlCNGC1*- and *SlCNGC14*-silenced leaves reduced expression of the negative defense regulatory gene *SlCAMTA3* by 2.5-fold, and generally increased expression of the positive defense regulatory genes *SlCaM6, SlCDPK10*, and *SlCBP60g* to different extent, which was much higher in *SlCNGC14*-silenced leaves than *SlCNGC1*-silenced leaves. Expression of *SlCaM6, SlCDPK10* and *SlCBP60g* genes in *SlCNGC1*-silenced leaves was 4.3-, 1.5- and 2.5-fold, respectively, as high as that in the eGFP controls, while these folds were 34.0, 4.5, and 9.1 for that in *SlCNGC14*-silenced leaves (**Figure 5**). This result implies that *SlCNGC1* and *SlCNGC14* might modulate the expression of these Ca^2+^ signaling genes.

## Discussion

We previously found that two tomato *CNGC* genes *SlCNGC1* and *SlCNGC14* play an important role in tomato HR elicited by the rice bacterial blight pathogen *X. oryzae* pv. *oryzae* (*Xoo*) (Saand et al., 2015a). Here, we further reveal that these two *CNGC* genes likely encode functional Ca^2+^ channels and negatively affect tomato non-host resistance to the rice bacterial leaf streak pathogen *Xoc*, through modulating ROS accumulation and callose deposition. The role of Ca^2+^ signaling pathway in non-host resistance to *Xoc* is previously unknown. Our results provide evidence for the contribution of *CNGC*-mediated Ca^2+^ signaling pathway to this non-host resistance.

In plants, only a few CNGCs such as AtCNGC2 and AtCNGC18 have been proved to function as Ca^2+^ channels (Gao et al., 2014; Wang et al., 2017). Whether the tomato *CNGC* genes encode functional Ca^2+^ channels remain to be verified. In this study, we demonstrated that silencing of *SlCNGC1* and *SlCNGC14* significantly reduced or almost abolished cytosolic Ca^2+^ accumulation (**Figure 4**). Thus, SlCNGC1 and SlCNGC14 likely function as Ca^2+^ channels. Further electrophysiological studies are required to provide more evidences to verify it.

It is recently reported that Arabidopsis *cngc2* mutant overaccumulates Ca^2+^ in apoplastic compartment and increased HR and resistance (Wang et al., 2017). Similarly, in this study, we found that silencing of *SlCNGC1* and *SlCNGC14* significantly reduced cytosolic Ca^2+^ accumulation and enhanced HR and non-host resistance to *Xoc* and PTI. Whether silencing of *SlCNGC1* and *SlCNGC14* results in overaccumulation of Ca^2+^ in apoplastic compartment as observed in *Atcngc2* mutant (Wang et al., 2017) requires further confirmation.

ROS is the well-known master signal in plant immunity and is indispensable to *Xoo*-induced HR and non-host resistance (Li et al., 2015). Thus, it is considerable that regulation of ROS accumulation represents one of the essential mechanisms underlying *SlCNGC1*- and *SlCNGC14*-dependent regulation of HR and non-host resistance to *Xoc* and PTI. How *SlCNGC1* and *SlCNGC14* negatively regulate ROS accumulation remains an intriguing question to be addressed. NADPH oxidase encoded by *RBOH* genes is the key enzyme to generate ROS during plant-pathogen interactions. Interestingly, Ca^2+^ affects the activity of this enzyme which contains EF-hands to bind Ca^2+^. Moreover, *AtCPK28*, a calcium-dependent protein kinase gene, negatively regulate ROS accumulation during PTI (Monaghan et al., 2014). In this context, it is notable that silencing of *SlCNGC1* and *SlCNGC14* strongly represses cytosolic Ca^2+^ accumulation and expression of a set of denfense-related Ca^2+^ signaling genes including *SlCaM6, SlCDPK10* and *SlCBP60g* (**Figures 4, 5**). Whether and how these changes affect NADPH oxidase thereby modulate ROS accumulation deserves further investigation.

SlCNGC1 and SlCNGC14 belong to group I and group III of tomato CNGC family. They function similarly in negative modulation of tomato HR and non-host resistance to *Xoc*. Both of them suppress ROS accumulation and callose deposition and alter expression of a same set of defense-related Ca^2+^ signaling genes including *SlCaM6, SlCDPK10* and *SlCBP60g*. However, the degree of effect in resistance responses by silencing SlCNGC1 and SlCNGC14 varies (**Figures 1–5**), indicating the difference of their involvement in this non-host resistance although it can be the consequence of difference of silencing degree. In animal, CNGC often forms homo- or hetero-oligomer to function (Kaupp and Seifert, 2002). Furthermore, some plant CNGC isoforms, such as AtCNGC2 and AtCNGC4, have been reported to be able to homo- or hetero-complex *in planta* (Chin et al., 2013). In this context, whether SlCNGC1 and SlCNGC14 form oligomer to regulate HR and plant immunity is worth further clarifying. Additionally, cellular localization of plant CNGCs other than long expected plasma membrane have been reported recently (DeFalco et al., 2016b). Whether the cellular localization and accumulation of SlCNGC1 and SlCNGC14 differ awaits further study.

Roles of CNGCs in plant disease resistance seem to be complex. Results from previous studies using Ca^2+^ channel blockers indicated that cytosolic Ca^2+^ elevation in response to PAMPs such as flg22 is required for PAMP induced defense resposnes including ROS burst (Jeworutzki et al., 2010; Ranf et al., 2011; Segonzac et al., 2011), suggesting a positive role of the possibly involved CNGCs in PTI defenses. However, CNGC genes likely play different roles in various types of resistance. For instance, T-DNA insertion knockout mutants for *AtCNGC11* and *AtCNGC12* exhibit unaffected flg22 induced PTI responses but show a partial breakdown of resistance against avirulent, but not virulent *Hyaloperonospora arabidopsidis* and *Pseudomonas syringae*, indicating that both *AtCNGC11* and *AtCNGC12* might be not involved in PTI but act as positive regulators of *R* gene-mediated resistance responses (Yoshioka et al., 2006; Moeder et al., 2011). Differently, null mutants for *AtCNGC2* and *AtCNGC4* display impaired HR cell death, while maintaining resistance against avirulent pathogens, exhibiting enhanced broad-spectrum resistance against virulent pathogens, elevated levels of SA, and constitutive expression of PR genes (Yu et al., 1998; Clough et al., 2000; Yu et al., 2000; Balagué et al., 2003; Jurkowski et al., 2004; Genger et al., 2008), and thus these AtCNGCs seem to play positive role in HR while have negative role in *R* gene-mediated resistance and basal resistance. Additionally, *AtCNGC2* was reported to positively contribute to Pep-elicited immunity (Qi et al., 2010; Ma et al., 2012, 2013) and LPS-triggered Ca^2+^ influx that led to nitric oxide production and consequent HR formation (Ali et al., 2007; Ma et al., 2007), but might be not involved in flg22-triggered immunity (Jeworutzki et al., 2010). In this study, we found the negative role of *SlCNGC1* and *SlCNGC14* in *Xoc*-induced HR and non-host resistance, adding further complexity to the function of plant CNGCs. It is likely that different members of CNGC family might have different roles in resistance and at least some CNGCs are multifunctional and differentially regulate various types of resistance against different pathogens. *SlCNGC1* and *SlCNGC14*, which exhibit similar function in *Xoc*-induced HR and non-host resistance, belong to different groups of CNGCs, with *SlCNGC1* to group I as *AtCNGC11* and *AtCNGC12*, while *SlCNGC14* to group III, indicating that the function of CNGCs is not group-dependent, as we suggested previously (Saand et al., 2015b). Mechanisms underlying plant CNGC-mediated resistance againt various pathogens await further elucidation.

## Conclusion

Gene silencing analyses demonstrated that *SlCNGC1* and *SlCNGC14* negatively regulate non-host resistance related HR cell death and non-host resistance to *X. oryzae* pv. *oryzicola* (*Xoc*) in tomato. These two *SlCNGCs* repress callose deposition and ROS accumulation to alleviate non-host resistance and likely also PTI. Silencing of *SlCNGC1* and *SlCNGC14* strongly reduced cytosolic Ca^2+^ accumulation, suggesting that SlCNGC1 and SlCNGC14 function as Ca^2+^ channels and negatively regulate non-host resistance and PTI through modulating cytosolic Ca^2+^ accumulation. *SlCNGC14* likely played a stronger regulatory role in the non-host resistance and PTI than *SlCNGC1*. Our results reveal the contribution of CNGC-mediated Ca^2+^ signaling pathway to non-host resistance and PTI.

## Author Contributions

The project was coordinated by X-ZC. X-RZ conducted the gene silencing analyses. X-RZ and Y-PX carried out the gene expression, designed and analyzed all statistical data. X-ZC conceived of the study, and participated in its design and coordination. X-ZC and X-RZ prepared the manuscript. All authors read and approved the final manuscript.

## Conflict of Interest Statement

The authors declare that the research was conducted in the absence of any commercial or financial relationships that could be construed as a potential conflict of interest.
