# Supplementary material for: SlCNGC1 and SlCNGC14 Suppress Xanthomonas oryzae pv. oryzicola-Induced Hypersensitive Response and Non-host Resistance in Tomato
Source: Front Plant Sci. 2018 Mar 6;9:285. doi: 10.3389/fpls.2018.00285 (PMC5845538; doi:10.3389/fpls.2018.00285)
Supplement: Supplementary file 1 [file Table_1.DOC]

**Supplementary Table S1 Primers used in this study**

| **Primer Name** | **Sequence (5'→ 3')** | **Enzyme site** |
| --- | --- | --- |
| **For expression analysis** |  |  |
| SlCNGC1-F | GAACACTGCAAGTCAGAAGA |  |
| SlCNGC1-R | TCACTTAGGAAAGGGGTGCA |  |
| SlCNGC14-F | CTCAACCGCTTCAAGAATTC |  |
| SlCNGC14-R | TAACCCAATAGATTGTCGGT |  |
| Sl 18SrDNA-F | GCCGGCGACGCATCATTCAAA |  |
| Sl 18SrDNA-R | CGCGCCTGCTGCCTTCCTT |  |
| SlCBP60g-F | ACCAGCAAACTTCACCTCCAT |  |
| SlCBP60g-R | ACATGCACCTTCATCCTGAC |  |
| SlCAMTA3-F | TGATGCATGAACAAGGAAAC |  |
| SlCAMTA3-R | TCAACTTCTGATCCATGGAC |  |
| SlCAM2-F | TAAACGAGGTGGATGCA |  |
| SlCAM2-R | CTCTGAATGCCTCTTTCAA |  |
| SlCAM6-F | AGAACTCAAAGAGGCTTTT |  |
| SlCAM6-R | AAGCATCATACGGACAAA |  |
| SlCDPK10-F | TATGGGGAATTCGCAACTA |  |
| SlCDPK10-R | CTATTGACCATTGTCACTG |  |
| **For VIGS analysis** |  |  |
| V-SlCNGC1-F | gcgaattcCTGCTCATCATCCACCTTCT | *Eco*RI |
| V-SlCNGC1-R | ttggatccCTGACTTGCAGTGTTCAAAC | *Bam*HI |
| V-SlCNGC14-F | gcgaattcAGATAGGTTACTTGTTGCTC | *Eco*RI |
| V-SlCNGC14-R | ttggatccGTCTGATATGCAATAGAACA | *Bam*HI |
